# Can Tumor-Infiltrating Lymphocytes (TILs) Be a Predictive Factor for Lymph Nodes Status in Both Early Stage and Locally Advanced Breast Cancer?

**DOI:** 10.3390/jcm8040545

**Published:** 2019-04-22

**Authors:** Alexandra Caziuc, Diana Schlanger, Giorgiana Amarinei, George Calin Dindelegan

**Affiliations:** First Surgical Clinic, University of Medicine and Pharmacy “Iuliu Hatieganu”, Victor Babes Street, no. 8, 400000 Cluj-Napoca, Romania; schlanger.diana@yahoo.com (D.S.); giorgianaamarinei@yahoo.com (G.A.); george.dindelegan@gmail.com (G.C.D.)

**Keywords:** breast cancer, TILs, lymph node, chemotherapy, pCR

## Abstract

The status of axillary lymph nodes is an important prognostic factor in the outcome of breast cancer tumors. New trials changed the attitude towards axillary clearance. In the era of development of new immune therapies for breast cancer, it is important to identify a biomarker that can predict lymph node status. Tumor-infiltrating lymphocytes (TILs) are a valuable indicator of the immune microenvironment that plays the central role in new anticancer drugs. Although the correlation between TILs and response to chemotherapy was established by previous studies, our retrospective study investigated the correlation between TILs and lymph node status. We analyzed data on 172 patients. According to stage, patients were divided in two groups: patients who underwent primary surgical treatment (breast-conserving or mastectomy and sentinel lymph node (SLN) biopsy +/− axillary clearance in conformity with lymph node status) and patients who received chemotherapy prior to surgical treatment (breast-conserving or mastectomy + axillary clearance). We showed a good inverse correlation between TILs and lymph nodes status for both early stage and locally advanced breast cancers. Moreover, TILs are a predictor for positive lymph nodes in the axilla in patients undergoing axillary clearance after SLN biopsy, with no statistical difference between the intrinsic or histological subtype of breast cancers. We also obtained a significant correlation between TILs and response to chemotherapy with no significative difference according to histological subtype. Although further data have still to be gathered before meeting the criteria for clinical utility, this study demonstrates that TILs are one of the most accredited forthcoming biomarkers for breast cancer (BC) patients.

## 1. Introduction

In the last three decades, treatment of breast cancer (BC) has changed remarkably, from invasive surgical interventions to breast-conserving surgery and new complementary and targeted therapies [1,2]. Development of radiotherapy regimens and techniques determined a new attitude towards the axilla, both in early stage and locally advanced tumors [3,4,5,6].

The immune tumor microenvironment (ITME) plays a central role in tailoring new immunologic therapies. Ongoing challenges remain in defining biomarkers that predict response to immunotherapy and the main characteristics of patients that can benefit from these new therapies [7]. Tumor-infiltrating lymphocytes (TILs) are a valuable indicator of ITME. Their functions can dynamically change during tumor progression [8,9]. Previous studies have shown their role in predicting disease-free and overall survival of patients with breast cancer and a good correlation with the patient’s response to chemotherapy. However, the results are contradictory regarding their association with different intrinsic subtypes of BC.

Tumors with high TILs may also have increased PD-L1 (programmed death-1 ligand) expression [10,11,12]. PD-L1 expression has been associated with higher histological grade, higher proliferation, hormonal receptor negativity, and higher TIL infiltration. Despite these associations several studies demonstrated a better outcome of patients with high expression of PD-L1 [13,14]. PD-L1 immune checkpoint inhibitors have been evaluated in clinical trials as new therapies for BC with favorable results [15,16].

The present study investigated the correlation between TILs and lymph nodes status in early stage and locally advanced BC. For those patients receiving neoadjuvant chemotherapy (NAC), we studied the correlation of TILs with the response to chemotherapy, evaluated by the number of cases who reached complete pathologic response (pCR) and the Miller–Payne (MPG) and Residual Cancer Burden (RCB) scales. As far as our knowledge, this is the first study to establish the predictive value of TILs for lymph nodes status, either sentinel or post axillary clearance.

## 2. Materials and Methods

### 2.1. Study Cohort

Our observational retrospective study enrolled patients newly diagnosed with early stage and locally advanced BC that were treated in our center between January 2016 and December 2018. Data collection for baseline variables was performed from October 2018 to January 2019, using electronic medical records. Approval from the ethics commission was obtained prior to data collection.

We excluded patients with inflammatory BC, stage IV disease, and without available pathology reports or incomplete data. Data on 172 patients were analyzed.

Information on age, menopausal status, tumor characteristics (size, grade, intrinsic molecular subtype, lymphovascular invasion, and TILs), treatment (NAC, type of surgical intervention), and outcomes (lymph nodes status, and response to NAC) were collected. The histopathological response to chemotherapy was evaluated on the surgical excision sample by MPG and RCB score.

#### 2.1.1. Type of Treatment

According to stage, patients were divided in two groups: primary surgical treatment (PST)-patients who underwent primary surgical treatment (breast-conserving therapy (BCT) or mastectomy and sentinel lymph node (SLN) biopsy +/− axillary clearance in conformity with lymph node status); and secondary surgical treatment (SST)-patients who received neoadjuvant chemotherapy (NAC) prior to surgical treatment (BCT or mastectomy + axillary clearance). NAC regimens consisted of 4 cycles of doxorubicin (60 mg/m^2^) and cyclophosphamide (600 mg/m^2^), followed by 4 cycles of docetaxel (75 mg/m^2^). Some patients received doxorubicin (60 mg/m^2^) plus docetaxel (75 mg/m^2^) or doxorubicin (60 mg/m^2^) plus cyclophosphamide (600 mg/m^2^) by intravenous infusion every 3 weeks for 6 cycles.

We evaluated separately the correlation between TILs and lymph node status in patients receiving chemotherapy and patients where we performed surgery and SLN biopsy. We considered positive nodes the presence of isolated tumor cells, micrometastases (cases in which we did not perform axillary clearance) and macrometastases (cases in which we performed axillary clearance).

#### 2.1.2. TILs Assessment

TILs were evaluated on core needle biopsies using hematoxylin and eosin staining, following the recommended consensus guideline from the International TILs Working Group [17,18]. The assessment of TILs was done by different pathologists within the routine analysis (one pathologist/ assessment per case). TILs levels were divided in four groups: 0—TILs < 10%, 1—10–40%, 2—40–60%, and 3—TILs > 60%.

### 2.2. Statistical Analysis

The statistical analysis was performed using EpiInfo version 7.2.2.6 (Centers for Disease Control and Prevention, Atlanta, Georgia). In both groups, we used an ANOVA test to evaluate the correlation between TILs and lymph nodes status. Moreover, we evaluated the correlation between TILs and lymphovascular invasion using ANOVA test. For the SST group, we investigated the correlation between TILs and the MPG, RCB, and pCR, as parameters of response to chemotherapy. For both lymph node status and response to chemotherapy, we evaluated the correlation with TILs according to the intrinsic and histological type of breast cancers using the Fisher test. A *p*-value under 0.05 was considered statistically significative.

## 3. Results

### 3.1. Baseline Characteristics

One hundred and seventy-two patients were included in our study (76 in the PST group, and 96 in the SST group), with a median age of 53.37 years (i.e., between the age of 30–76 years old). Baseline characteristics of the patients are presented in Table 1. Most of the cases receiving PST were luminal A, while cases receiving SST were locally advanced tumors luminal A and triple negative. Ductal histological subtype was the most frequent (76.31% in the PST group, and 91.66% in the SST group), with only 8 cases of mucinous tumors in both groups (4.65%). Most tumors (77.90%) were estrogen-positive, with Ki-67 over 20% in 48.25% of the cases.

### 3.2. PST Group

**Figure 1:**
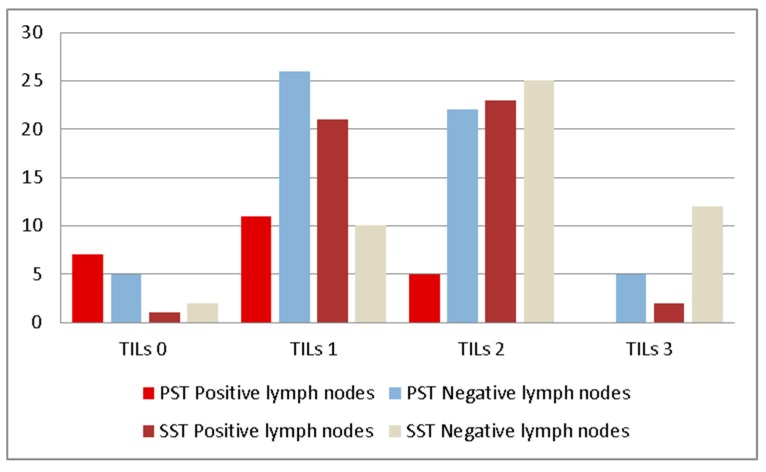
Lymph nodes status according to TILs subgroups.

Statistical analysis showed a significative correlation between TILs and SLN status (*p* = 0.02) in the PST group (Table 2, Figure 1). Moreover, when we evaluated the correlation between TILs and the lymph nodes status after axillary clearance (or axillary lymph node dissection (ALND)) performed for patients with macrometastases, *p* was also significative (*p* = 0.01, Table 2). This correlation was not influenced by the intrinsic (*p* = 0.32, Fisher test) nor histological (*p* = 0.89, Fisher test) subtype of the tumor.

According to our analysis, the correlation between TILs and lymphovascular invasion was not statistical significative for the PST group (*p* = 0.89).

### 3.3. SST Group

Our analysis showed a significative correlation (Table 3, Figure 1) between TILs and lymph node status after ALND for patients that received NAC (*p* = 0.01). This significative correlation was observed also for MPG (*p* = 0.002) and RCB (*p* = 0.02), as you can see in Table 4.

Moreover, we found a positive statistical correlation between TILs and pCR (*p* = 0.001, Table 4), suggesting that elevated number of TILs can predict a complete response to chemotherapy.

Our analysis did not show statistical differences (*p* = 0.49, Fisher test) in the correlation between lymph nodes status and TILs according to the intrinsic subtype of tumors. We did not find any statistical difference according to histological subtype in the correlation of TILs with the lymph node status (*p* = 0.55, Fisher test) and the response to chemotherapy (*p* = 0.40, Fisher test). However, the capacity of predicting response to chemotherapy was significative for triple negative tumors (*p* = 0.03, Fisher test for RCB, and *p* = 0.04, Fisher test for MPG) and HER2-positive tumors (*p* = 0.04, Fisher test for RCB, and *p* = 0.03, Fisher test for MPG) and not significative for luminal A and luminal B HER2 negative tumors (*p* > 0.05, Fisher test for both RCB and MPG). Regarding the correlation between TILs and lymphovascular invasion, the *p*-value was 0.03.

## 4. Discussion

Our study showed a good correlation between TILs and lymph nodes status for both early stage and locally advanced breast cancers. Moreover, TILs were a predictor for positive lymph nodes in the axilla in patients undergoing ALND after a SLN biopsy, with no statistical difference between the intrinsic or histological subtype of breast cancers. We obtained a significant correlation between TILS and response to chemotherapy (either evaluated by RCB or MPG, or considering only pCR) in the SST group. Although we did not find any significative difference according to histological subtype, the correlation was statistically significative only for triple negative and HER2-enriched tumors when considering intrinsic subtypes of breast cancer.

Many studies recognized the importance of the immune tumor microenvironment in breast cancer [19], both as an important element for tumor development and progression, and as parameter of response to treatment becoming one of the new therapeutic targets. TILs are the key players of ITME. The first association between breast cancer and TILs was made in 1992, suggesting that a rich lymphocytes infiltrate can predict recurrence-free survival [20] in breast cancer tumors with a high proliferation rate. Since then, the importance of TILs in breast cancer has been demonstrated by various studies.

However, one of the main issues when taking into consideration TILs remains to standardize assessment and to maximize reproducibility [21]. The International Immuno-Oncology Biomarker Working Group on Breast Cancer developed criteria for TILs assessment that decrease inter-observer variability [22]. These criteria define TILs as intraepithelial mononuclear cells within tumor cell nests or in direct contact with tumor cells; stromal TILs are lymphocytes in the tumor stroma without direct contact with tumor cells. TILs should be assessed as a continuous parameter on a single hematoxylin and eosin-stained tumor section [17,23]. Our study used these criteria for assessment, although we preferred to study the correlation between TILs and the established parameters by considering four groups: TILs < 10%, 10–40%, 40–60%, and TILs > 60%. We believe that this approach could eliminate the possible bias given by inter-observer variability since the assessment was done by different pathologists. As our study was retrospective, the pathologists involved in TILs assessment were not specially trained for the project. Moreover other studies that analyzed the capacity of TILs to predict the status of lymph nodes in melanoma or gastric cancer used a similar division. The latest studies in breast cancer immunology describe a special subtype, lymphocytic-predominant, with the highest TILs infiltration (50–60% lymphocytic infiltration of either tumor epithelium or stroma). Although this subgroup included a lower number of patients, it was associated with the best outcome [24,25].

Another issue is to establish the most suitable tissue sample for TILs assessment, since previous studies showed significative inconsistencies between core needle biopsies and surgical tissue samples [26] and, furthermore, between pre- and post-NAC [27]. All our assessments were done on tissue samples from core needle biopsies. Further studies should, however, investigate the effect of changes in TILs rates after chemotherapy over disease-free survival and overall survival, since the preliminary results showed a better outcome for patients who after chemotherapy achieved higher levels of TILs [28].

Previous studies correlated high TILs levels with a better response to NAC. While some demonstrated this correlation only for aggressive intrinsic subtypes (triple negative and HER- enriched) [29,30], others stated that the rate of achieving pCR is equal in all intrinsic subtypes [31,32]. However, studies confirmed that disease-free survival and overall survival are influenced by high levels of TILs only for triple negative [30] and HER2-enriched [33]. No statistical difference was found between the chemotherapy regimens [34]. Our study confirmed the results regarding the correlation between TILs and response to NAC for triple negative and HERr2-enriched breast tumors, both for pCR, and for MPG and RCB scores. This data showed that not only pCR can be correlated to TILs. We included MPG and RCB scores in the study because they are also predictors for disease-free and especially for overall survival [35,36,37]. Asano et al. imagined RCB-TILs score that could be predictive for overall survival after NAC [38].

The correlation between increased TILs and lymph node status was first stated for early gastric cancer [39] and melanoma [24]. As far as our knowledge, our study is the only one that established a correlation between TILs and lymph node status for both early and locally advanced breast tumors. Attempts to correlate TILs with lymph nodes status in triple negative tumors have failed [40]. Since the attitude versus BCT, and especially towards the axilla, changed significative in the last years after the published results of AMAROS clinical trial [41,42], the importance of identifying low and high risk-profile tumors has increased.

In our study, an increased number of TILs was associated with a lower number of lymph nodes metastases both in PST and SST groups. For early stage tumors for TILs over 60%, all lymph nodes were negative, with only 2 positive lymph nodes in the SST group, suggesting that lymphocytic predominant breast cancers have the lowest aggressivity. TILs can be considered valuable biomarkers that can help tailoring suitable therapies and improving the stratification of BC patients for immune-based treatment selection.

Previous studies demonstrated the correlation between lymphovascular invasion and positive lymph nodes in the axilla [43]. Also, when present, lymphovascular invasion correlates with poor prognosis regarding survival and response to chemotherapy [44,45]. Our study investigated the correlation between TILs and lymphovascular invasion. We only found a statistical significative correlation for locally advanced BC. However, our results could be biased by the relatively small sample of patients that were positive for lymphovascular invasion in the PST group.

The magnitude of TILs is variable within and between breast cancer subtypes [29]. Most studies [19] correlated high TILs with high risk subtypes of cancers (HER2-enriched and triple negative). For patients with BRCA 1 and 2 mutations, TILs proved itself to be a favorable factor of disease-free survival. For overall survival, for each 10% increase of TILs, it was demonstrated a 10% reduction in mortality for BRCA 1 carriers, with no significative effect on BRCA 2 mutation patients [46]. We demonstrated the predictive value of TILs in lymph nodes status for all intrinsic subtypes, with significative differences regarding only the response to NAC.

Our results showed the importance of TILs (as an indicator of ITME) in the outcome of early stage and locally advanced breast cancers. Previous studies demonstrated, however, that ITME can be influenced by multiple factors, such as comorbidities, drugs, and smoking [47]. ITME can also be influenced during chemotherapy, and these changes could also be considered important predictive factors for the outcome of breast cancer patients [7]. We can assume that TILs also modify, according to these factors. Our study did not follow these possible secondary factors. Further studies should investigate their control over ITME activation and their effect over TILs.

There are many reports on the utility of TILs as prognostic factors and effect predictors, but until present, these findings were not used in the clinical management of the patients. Although our study is limited by its retrospective nature and the relatively small samples studied, it demonstrated the possibility of using TILs as a biomarker in the clinical setting for prediction in lymph nodes status.

## 5. Conclusions

Although further data have still to be gathered before meeting the criteria of clinical utility, this study demonstrated that TILs are useful predictive factors for lymph nodes status and response to NAC for both early stage and locally advanced BC patients, as well as sustain the idea of TILs as one of the most accredited forthcoming biomarkers for BC patients.

## Figures and Tables

**Table 1:** Patients characteristics in the two groups.

		Group PST (*n* = 76)	Group SST (*n* = 96)
Age–median (range) in years		51.81 (30–73)	54.93 (27–76)
Tumor size (cT)	T1	57 (75.00%)	8 (8.33%)
	T2	19 (25.00%)	42 (43.75%)
	T3	-	25 (26.04%)
	T4	-	21 (21.87%)
Lymph node status (cN)	N0	76 (100%)	3 (3.12%)
	N1	-	65 (67.70%)
	N2	-	25 (26.04%)
	N3	-	3 (3.12%)
Histological subtype	Ductal	58 (76.31%)	88 (91.66%)
	Lobular	4 (5.26%)	3 (3.12%)
	Mucinous	4 (5.26%)	4 (4.16%)
	Other	10 (13.15%)	1 (1.04%)
Estrogen receptors positivity		72 (94.73%	62 (64.58%)
Progesterone receptor positivity		64 (84.21%)	52 (54.16%)
HER2 positivity		11 (14.47%)	29 (30.20%)
Ki67 > 20%		23 (30.26%)	60 (62.50%)
Intrinsic subtype (IHC4)	Luminal A	45 (59.21%)	23 (23.95%)
	Luminal B HER2 -	16 (21.05%)	19 (19.79%)
	Luminal B HER2+	11 (14.47%)	20 (20.83%)
	HER2 over expression	0 (0.00%)	9 (9.37%)
	Triple negative	4 (5.26%)	25 (26.04%)
Lymphovascular invasion		10 (13.15%)	36 (37.50%
TILs	0	12 (15.78%)	3 (3.12%)
	1	37 (48.68%)	31 (32.29%)
	2	22 (28.94%)	48 (50.00%)
	3	5 (6.57%)	14 (14.58%)

PST (primary surgical treatment), patients who underwent primary surgical treatment (breast-conserving therapy (BCT) or mastectomy and sentinel lymph node (SLN) biopsy +/− axillary clearance in conformity with lymph node status); SST (secondary surgical treatment), patients who received neoadjuvant chemotherapy (NAC) prior to surgical treatment (BCT or mastectomy + axillary clearance); TILs, tumor-infiltrating lymphocytes.

**Table 2:** Lymph nodes status according TILs values in the PST group.

TILs	Positive SLN/no. of Cases *n* (%)	Type of Metastasis	Positive Nodes after ALND/ no. of Cases *n* (%)
0	7/12 (58.33%)	5M, 1m, 1 ITC	3/12 (25.00%)
1	11/37 (29.72%)	9M, 1 m, 1 ITC	3/37 (8.10%)
2	5/22 (22.72%)	4M, 1 m	0/22 (0.00%)
3	0/5 (0.00%)	-	-

ITC, isolated tumor cells; m, micrometastasis; M, macrometastasis; SLN, sentinel lymph nodes; ALND, axillary lymph node dissection.

**Table 3:** Lymph nodes status according to TILs values in the SST group.

TILs	Positive Nodes after ALND/ no. of Cases *n* (%)	Type of Metastasis
0	1/3 (33.33%)	1M
1	21/31 (67.74%)	20M, 1 m
2	23/48 (47.91%)	20M, 2 m, 1 ITC
3	2/14 (14.28%)	1M, 1 m

**Table 4:** Tumoral response to chemotherapy according to TILs values.

TILs	pCR/no. of Cases *n* (%)	MPG (Mean)	RCB (Mean)
0	1/3 (33.33%)	4	1.33
1	7/31 (22.58%)	3.03	2.25
2	7/48 (14.58%)	3.22	1.79
3	10/14 (71.42%)	4.42	0.50

pCR, complete pathologic response; MPG, Miller–Payne grade; RCB, Residual Cancer Burden score.
